# Validation of dot blot hybridization and denaturing high performance liquid chromatography as reliable methods for *TP53 *codon 72 genotyping in molecular epidemiologic studies

**DOI:** 10.1186/1471-2156-11-44

**Published:** 2010-05-26

**Authors:** Tatiana Rabachini, Helen Trottier, Eduardo L Franco, Luisa L Villa

**Affiliations:** 1 Ludwig Institute for Cancer Research, São Paulo, Brazil; 2 Hospital Alemão Oswaldo Cruz. R. João Julião, 245, 1° andar, Cep. 01323-903, São Paulo (SP), Brazil; 3 Instituto de Química, Universidade de São Paulo, Brazil; 4 Av. Prof. Lineu Prestes, 748, Butantã, Cep. 05508-900, São Paulo (SP), Brazil; 5 Departments of Oncology and Epidemiology, McGill University.546 Pine Ave W, Montreal, Quebec H2W1S6, Canada; 6 Department of Social and Preventive Medicine and Ste-Justine Hospital, University of Montreal, 3175 Côte Ste-Catherine, Montreal, Quebec H3T 1C5, Canada

## Abstract

**Background:**

Mutations in *TP53 *are common events during carcinogenesis. In addition to gene mutations, several reports have focused on *TP53 *polymorphisms as risk factors for malignant disease. Many studies have highlighted that the status of the *TP53 *codon 72 polymorphism could influence cancer susceptibility. However, the results have been inconsistent and various methodological features can contribute to departures from Hardy-Weinberg equilibrium, a condition that may influence the disease risk estimates. The most widely accepted method of detecting genotyping error is to confirm genotypes by sequencing and/or via a separate method.

**Results:**

We developed two new genotyping methods for *TP53 *codon 72 polymorphism detection: Denaturing High Performance Liquid Chromatography (DHPLC) and Dot Blot hybridization. These methods were compared with Restriction Fragment Length Polymorphism (RFLP) using two different restriction enzymes. We observed high agreement among all methodologies assayed. Dot-blot hybridization and DHPLC results were more highly concordant with each other than when either of these methods was compared with RFLP.

**Conclusions:**

Although variations may occur, our results indicate that DHPLC and Dot Blot hybridization can be used as reliable screening methods for *TP53 *codon 72 polymorphism detection, especially in molecular epidemiologic studies, where high throughput methodologies are required.

## Background

The tumour suppressor protein p53 is a key transcription factor that participates in numerous homeostatic functions such as cell cycle checkpoint control, repair of DNA damage and apoptosis induction [1]. Tumour-associated mutations in TP53, typically single nucleotide substitutions in the coding sequence, are a hallmark of most human cancers and cause dramatic defects in p53 function [2]. By contrast, only a small fraction of the naturally occurring sequence variations (single nucleotide polymorphisms, SNP) of *TP53 *in human populations are expected to cause measurable perturbation of p53 function [3]. The best studied *TP53 *SNP is on codon 72. In human populations, codon 72 of *TP53 *has either the sequence CCC, which encodes proline, or CGC, which encodes arginine. *TP53 *codon 72 polymorphism alters the ability of the p53 protein to induce apoptosis, influences the behaviour of mutant p53 and decreases DNA repair capacity [4-6] .

The role of this polymorphism in carcinogenesis has been well studied but is a matter of controversy. A decade ago, a dramatic effect of the *TP53 *codon 72 polymorphism on the risk of cervical cancer was reported. This effect was explained by the finding that the E6 oncoprotein from high-risk mucosal human papillomaviruses (HPVs) causes more efficient degradation of the Arg form of the p53 protein than of the Pro form, thus reducing cellular levels of p53 protein and increasing the risk of HPV-associated cancers in TP53-Arg homozygotes [7]. This sparked an intensive investigation into the potential effect of *TP53 *codon 72 polymorphism on susceptibility to cervical cancer or cancer-related phenotypes. Many of these studies have reported significant associations. However, the inconsistency in findings suggests that results should be interpreted with caution. For cervical cancer, a meta-regression analysis identified that the most important factor contributing to heterogeneity among results for invasive lesions was departure from Hardy-Weinberg equilibrium in the control group. However, various methodological features can contribute to departures from Hardy-Weinberg equilibrium and consequently to less than ideal circumstances for the examination of this polymorphism [8]. Therefore, confirming genotype results with a second method can reduce misclassification of genotype [9]. In doing so, deviations from Hardy-Weinberg equilibrium can be avoided. With this objective in mind, we aimed to compare different methodologies to define reference-quality methods for *TP53 *codon 72 genotyping. We developed two new methods for *TP53 *codon 72 polymorphism detection: Denaturing High Performance Liquid Chromatography (DHPLC) and Dot Blot hybridization. These methods were compared with Restriction Fragment Length Polymorphism (RFLP), a well establish method for *TP53 *codon 72 polymorphism genotyping, using two different restriction enzymes. We report herein the findings obtained with the validation of these methods and propose that they could be used for *TP53 *codon 72 polymorphism detection in molecular epidemiologic studies.

## Results

Using a combination of both PCR strategies described above we were able to amplify *TP53 *exon 4 of 962 samples, approximately 98.3% of all subjects tested. Samples were submitted to *TP53 *codon 72 genotyping using four different methods: DHPLC, Dot Blot hybridization and RFLP using two different restriction enzymes(*Bsa*JI and *Bst*UI).

**Figure 1:**
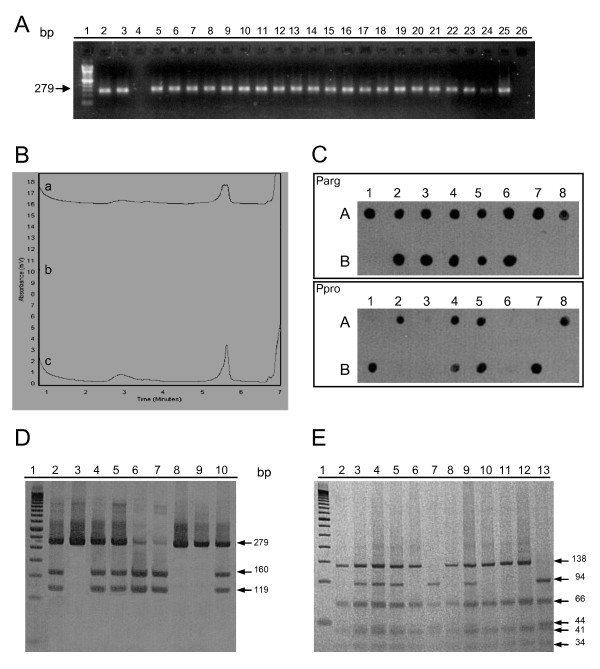
**p53 codon 72 genotyping**. (A) PCR for *TP53 *exon 4 detection. PCR products were loaded on 1.5% agarose gel. 100 base pairs DNA ladder (lane 1), positive controls (lane 2 and 3), negative controls (lanes 4 and 26), samples tested (lanes 5-25). (B) Detection of *TP53 *codon 72 polymorphism by DHPLC. Elution profiles obtained at 59°C. Representative heterozygous (a) and homozygous profile (b and c) are depicted. Note the small shoulder only in heterozygous samples. After the first trial, homozygous samples were mixed in approximately equimolar proportions with a control sample with *TP53 *codon 72 polymorphism previously identified as homozygous proline. This allowed differentiating the two homozygous genotypes. Homozygous profiles represent a sample with the genotype similar to the control sample added. Heterozygous profile represents a sample with the genotype differing from the control sample. (C) Dot Blot hybridization for *TP53 *codon 72 polymorphism genotyping. Samples were spotted onto nylon membranes and hybridized with biotin-labelled oligonucleotide probes for allele Arg (PArg) and allele Pro (PPro). Sample 1A, 1B and 2A represent positive controls. The profile observed in sample 1A is compatible with a homozygous arginine. Sample 1B presents a profile compatible with the genotype homozygous proline. Sample 2A presents a profile compatible with the heterozygous genotype. (D) Silver-stained 8% polyacrylamide gel showing the restriction profiles obtained with enzyme *Bst*UI of the *TP53 *exon 4 PCR product. 50 base pairs DNA ladder (lane 1). Lanes 2, 4, 5 represent heterozygous samples where fragments of 279, 160 and 119 base pairs can be detected. Lanes 3 and 8 represent homozygous proline samples in which the enzyme was not able to digest the PCR product. Lanes 6 represent an homozygous arginine in which fragments of 160 and 119 base pairs can be identified. Lanes 7, 9 and 10 represent positive controls for homozygous arginine, homozygous proline and heterozygous samples, respectively. (E) Silver-stained 12% polyacrylamide gel showing the restriction profiles obtained with enzyme *Bsa*JI of the *TP53 *exon 4 PCR product. 50 base pairs DNA ladder (lane 1). Lanes 3, 4, 5 represent heterozygous samples in which fragments of 138, 94, 66, 44, 41 and 34 base pairs can be detected. Lanes 7 represent a homozygous proline sample in which fragments of 94, 66, 44, 41 and 34 base pairs can be identified. Lanes 2, 6, 8, 10 and 11 represent homozygous arginine samples in which fragments of 138, 66, 41 and 34 base pairs can be detected. Lanes 9, 12 and 13 represent positive controls for heterozygous, homozygous arginine and homozygous proline samples, respectively.

DHPLC analysis of *TP53 *exon 4 at 59°C revealed a small shoulder only in heterozygous samples. The first assessment allowed the distinction of homozygous and heterozygous samples, as illustrated in Figure 1B. We were able to identify that 428 samples (44.5%) were heterozygous for *TP53 *codon 72 polymorphism. The second assessment which included a previously known PCR product in the assays revealed that 377 (39.2%) of samples were homozygous Arg/Arg and 157 (16.3%) were homozygous Pro/Pro (Table 1). Once the same profile was observed, we concluded that samples with the same genotype of the PCR product added presented a single peak, while samples with a different genotype of this added product presented a small shoulder, indicating heteroduplex formation.

**Table 1:** Frequencies of *TP53 *codon 72 genotypes for 962 women who were tested by 4 methods

Typing method	Genotype frequencies (%)		
	
	Arg/Arg	Pro/Arg	Pro/Pro
DHPLC	377 (39.2)	428 (44.5)	157 (16.3)
Dot Blot	374 (38.9)	418 (43.4)	170 (17.7)
RFLP- *Bsa*JI	375 (39.0)	437 (45.4)	150 (15.6)
RFLP-*Bst*UI	319 (33.2)	462 (48.0)	181 (18.8)

Using Dot-blot hybridization we observed that 418 samples (43.4%) were heterozygous for *TP53 *codon 72 polymorphism (Table 1). The hybridization patterns observed both for heterozygous and homozygous samples are shown in Figure 1C. Homozygous Arg/Arg represented 374 (38.9%) samples while homozygous Pro/Pro represented 170 (17.7%) of all samples tested.

*TP53 *exon 4 was also subjected to RFLP using the restriction enzymes *Bsa*JI and *Bst*UI. For both enzymes a unique digestion profile is obtained when *TP53 *exon 4 alleles codes for proline or arginine. *Bsa*JI leads to generation of five fragments when *TP53 *codon 72 encodes the amino acid proline (94, 66, 44, 41 and 34 base pairs). However, only four fragments are obtained when *TP53 *codon 72 encodes the amino acid arginine (138, 66, 41 and 34 base pairs). Heterozygous forms present a digestion profile representative of both alleles (Figure 1D). *Bst*UI leads to generation of two fragments when *TP53 *codon 72 encodes for arginine (160 and 119 base pairs). However, this enzyme is not able to cleave *TP53 *encoding homozygous proline in the codon 72. Heterozygous forms present a digestion profile representative of both alleles (Figure 1E).

Using *Bsa*JI we observed that 437 samples (45.4%) were heterozygous for *TP53 *codon 72 polymorphism (Table 1). Homozygous Arg/Arg represented 375 (39%) samples while homozygous Pro/Pro represented 150 (15.6%) of all samples tested. Using *Bst*UI we identify 462 (48%) heterozygous samples. A total of 319 (33.2%) were genotyped as homozygous Arg/Arg and 181 (18.8%) were genotyped as homozygous Pro/Pro (Table 1).

The agreement between methods is summarized in table 2. We observed high agreement in all combinations tested. Dot-blot hybridization and DHPLC results were more highly concordant with each other (kappa coefficient 0.92). Of the total of 962 samples analyzed, only 43 (4.5%) presented discordant results. The higher discordance was observed between DHPLC and RFLP using *Bst*UI (kappa coefficient 0.83). We observed disagreement between 102 samples, approximately 10% of all samples tested. The comparison between RFLP using *Bst*UI with all methods tested revealed that this enzyme may lead to misclassification. However, this disagreement was not sufficient to lead to departure from Hardy-Weinberg equilibrium. With all methods tested, the majority of samples were genotyped as heterozygous, followed by a high percentage of homozygous arginine and a lower percentage of homozygous proline.

**Table 2:** Inter-method variation in *TP53 *genotyping

Typing method	Frequency of joint distribution (%)		Weighted Kappa Coefficient
	
	Agreement	Disagreement	
DHPLC × Dot Blot	919 (95.5)	43 (4.5)	0.92
DHPLC × *Bsa*JI	887 (92.2)	75 (7.8)	0.88
DHPLC × *Bst*UI	860 (89.4)	102 (10.6)	0.83
Dot Blot × *Bsa*JI	895 (93.0)	67 (7.0)	0.90
Dot Blot × *Bst*UI	864 (89.8)	98 (10.2)	0.85
*Bsa*JI × *Bst*UI	869 (90.3)	93 (9.7)	0.85

## Discussion

Traditional studies that investigated individual *TP53 *polymorphisms in case-control studies of limited size have not given definitive answers and new approaches are required. High-throughput methodologies and consortium studies investigating large number of individuals may provide the robustness these investigations require while minimizing genotyping errors [3].

For cervical cancer, a meta-analysis identified that the most important factor contributing to heterogeneity among results for invasive lesions was departure from Hardy-Weinberg equilibrium in the control group [8]. According to these authors, various methodological features can contribute to departures from Hardy-Weinberg equilibrium and consequently to less than ideal circumstances for the examination of this polymorphism. To avoid these pitfalls, confirming genotype results with a second method can significantly reduce genotyping misclassification [9].

The results presented in this report indicate that DHPLC and Dot Blot can be used as reliable methods to identify *TP53 *codon 72 polymorphism. The comparison between these methods and RFLP, a well established methodology, indicate that Dot-blot and DHPLC results are more highly concordant with each other than when either of these methods was compared with RFLP only.

RFLP analysis was one of the first techniques to be widely used for detecting variation at the DNA sequence level [10]. The principle behind the technology rests on the possibility of comparing band profiles generated after restriction enzyme digestion in DNA molecules of different individuals. Diverse restriction sites that might have occurred affect DNA molecules in different ways, producing fragments of variable lengths. These differences in fragment lengths can be seen after gel electrophoresis [11]. However, several conditions need to be optimized in order to achieve the high sensitivity (the amount of DNA, the time for complete digestion, acrylamide concentration, etc). Additionally, if the DNA fragment is only partially digested the interpretation of band patterns may lead to misclassification. Compared with RFLP, dot-blot hybridization can be considered a moderately demanding technique. However, Dot blot is much less time consuming since a large number of samples can be hybridized on a single membrane. On the other hand, DHPLC can be considered a quick and easy screening method. This method allows the identification of mutations and polymorphisms based on detection of heteroduplex formation between mismatched nucleotides in double stranded PCR amplified DNA. Sequence variation creates a mixed population of heteroduplexes and homoduplexes during reannealing of heterozygous and homozygous DNA. When this mixed population is analyzed by HPLC under partially denaturing temperatures, the heteroduplexes elute from the column earlier than the homoduplexes because of their reduced melting temperature [12]. Thus, the sensitivity of this method is dependent on the temperature at which the analysis is completed. For the analysis of *TP53 *codon 72 polymorphism we have relied on the software-based predictions and we also selected overlapping temperatures to increase the chance of heteroduplex detection. Although we have not detected any false negative or false positive cases using DHPLC and Dot Blot, it is advisable to sequence a small percentage of randomly selected samples as a standard procedure for quality control purposes.

The high concordance between DHPLC and Dot Blot reveal that both methodologies are sensitive, reliable, fast and reproducible. Our results demonstrate that these methods can be used in combination with each other or in combination with RFLP*Bsa*JI and RFLP-*Bst*UI to specifically detect *TP53 *codon 72 polymorphism. Even with some level of disagreement, observed when RFLP-*Bst*UI was used as genotyping method, we did not observe departure from Hardy-Weinberg equilibrium, one of the most important factors contributing to disagreement observed in the literature.

## Conclusions

Because *TP53 *codon 72 polymorphism has been associated with cancer susceptibility, prognosis, response to treatment, and even survival it is of fundamental importance to efficiently and accurately detect this polymorphism in biological specimens. The use of appropriate methods may have a remarkable importance for defining the real role of *TP53 *codon 72 polymorphism in many types of human neoplasias.

## Methods

### Samples

We tested for *TP53 *codon 72 polymorphism specimens from a sub-cohort of 978 Brazilian women enrolled in the Ludwig-McGill cohort study [13]. The Ludwig-McGill Cohort Study is an ongoing investigation of the natural history of HPV infection and the risk of cervical neoplasia. This study adhered to the tenets of the Declaration of Helsinki. All participants entered the study only after giving signed informed consent. All study procedures and the informed consent were approved by the institutional review boards and ethical committees of the Ludwig Institute for Cancer Research and the Maternidade Escola Vila Nova Cachoeirinha clinic, both in São Paulo, Brazil [13]. DNA was extracted from exfoliated cervical cells and purified by spin column chromatography. Four different methodologies were used to detect *TP53 *codon 72 polymorphism: DHPLC, RFLP using two different restriction enzymes - *Bst*UI and *Bsa*JI and Dot-blot hybridization. Because *TP53 *codon 72 polymorphism is located at *TP53 *exon 4, previous amplification of this region was performed. The resultant amplicon was then tested by the methodologies described below.

### Amplification of *TP53 *exon 4

To establish and optimize *TP53 *codon 72 genotyping we performed a previous amplification of *TP53 *exon 4. Since this exon encodes a polyproline domain [14] GC-rich regions are frequently found. This condition favoured the generation of primer-dimers during amplification leading to low yield of PCR product. To avoid this problem we used hot start PCR to increase specificity [15]. In addition, because optimal primers annealing temperatures differ for more than 6°C we also used touchdown PCR to prevent non-specific extensions. The combination of both PCR strategies improved the yield of amplification and a single fragment of 279 base pairs was obtained (Figure 1A). The total reaction volume for DNA amplification was 50 μL. Each reaction was carried out using 0.5 μM of each of the two primers: *TP53 *+, 5'-TCCCCCTTGCCGTCCCAAG-3'; and *TP53 *-, 5'-CGTGCAAGTCACAGACTT-3'; in a mixture containing 10 mM Tris-HCl (pH 8.5), 50 mM KCl, 1.5 mM MgCl_2_, 200 mM of each deoxiribonucleotides, and 2 U of Taq Gold DNA Polymerase (Applied Biosystems -- Roche, New Jersey, NY). All other reagents were purchased from GIBCO BRL (Gaithersburg, MD). One microliter of DNA was added to the reaction. A negative control containing no DNA was also included with each series of reactions to check for contamination. For controls, we included 50 ng of DNA from SW756 cells and from a penile condyloma, known to be heterozygous and homozygous *TP53 *-Pro in codon 72, respectively. Reactions were conducted in a TC-341 thermal controller (Amersham Pharmacia Biotech, Buckinghamshire, England) with the following amplification profile: The following amplification profile was employed: 95°C for 10 min, 10 cycles of 95°C for 1 min, 58°C for 1 min and 72°C for 1 min followed by 15 cycles of 95°C for 1 min, 56°C for 1 min and 72°C for 1 min and an additional round of 15 cycles of 95°C for 1 min, 53°C for 1 min and 72°C for 1 min. A final extension step was added including 72°C for 3 min followed by 95°C for 10 min. Amplification products were run on 1.5% agarose gels containing 3 mg/ml of ethidium bromide at 90--100 V for 20--30 min. Bands were visualized and photographed on a UV transilluminator. Positive reactions were selected for *TP53 *codon 72 polymorphism detection.

### Denaturing HPLC Analysis (DHPLC)

PCR products were heated at 95°C for 10 min and allowed to cool down to room temperature for approximately 30 min. Five to 10 μL of each sample were run on a Wave DNA Fragment Analysis System (Transgenomic, Omaha, NE) using a DNASep column and the run was monitored by ultraviolet light (UV) (260 nm). Optimum DHPLC temperatures were determined by an incremental temperature scan, using the software-predicted melting profile as a starting point (Transgenomic, Omaha, NE). Samples were run at more than one temperature due to the heterogeneity in the distribution of GC-rich regions that results in a heterogeneous melting temperature distribution throughout the amplified DNA fragments to be analyzed. By using 59°C as a melting temperature we were able to identify the formation of heteroduplexes in a subset of samples submitted to the analysis. The heteroduplexes are frequently found in samples with differences in alleles. Thus, heterozygous samples were identified due to a small shoulder peak in the chromatogram. After distinguishing the heterozygous samples, the subset of homozygous samples were mixed in approximately equimolar proportions with a control sample with *TP53 *codon 72 polymorphism previously identified as homozygous proline. This mixture was heated at 95°C for 10 min and allowed to cool down to room temperature for approximately 30 min. Five to 10 μL of each sample were then submitted to a new DHPLC analysis. This allowed differentiating the two homozygous genotypes. Heteroduplexes were observed in samples with genotype homozygous arginine, since the PCR product added to the sample was known to be homozygous proline. The homoduplexes, or samples with a single peak, were observed in samples with genotype proline, since it was the same genotype presented by the control sample added to the mixture. As an additional control, 10% of the samples were randomly selected and mixed with the same equimolar proportions of a control sample previously identified as homozygous arginine. Results were compared and no discordant results were observed between the two pulled analyses.

### Dot Blot Hybridization

After amplification of *TP53 *gene exon 4, 1.5 μL of PCR products were spotted on nylon membranes (Hybond N^+ ^- Amersham Pharmacia Biotech, Buckinghamshire, England) and UV-cross-linked. Dot-blotted PCR products were hybridized with sequence-specific 3'; biotin-labelled oligonucleotide probes *TP53 *Arg 5'--GGGCCACGCGGGGAGCA-3' and *TP53 *Pro 5'--GGGCCACGGGGGGAGCA- 3'. Hybridization was performed in two different temperatures: 62°C for *TP53 *Arg and 64°C for *TP53 *Pro probe. Briefly, membranes were blocked with 15 ml 0.1 × SSPE (18 mM NaCl; 1 mM NaH_2_PO_4_-H_2_O; 0.55 mM NaOH; 0.1 mM EDTA; adjusted for pH 7,4), 0.5% SDS for 20 min and then pre-hybridized with 15 ml of 2 × SSPE (360 mM NaCl; 20 mM NaH_2_PO_4_-H_2_O; 11 mM NaOH; 2 mM EDTA; adjusted for pH 7,4), 0.1% SDS for 20 min. All steps above were performed in probe-specific hybridization temperature. Probes were added to pre-hybridization solution to a final concentration of 1.2 pmoles/mL. After overnight hybridization, membranes were washed 2 times of 15 min with 2 × SSPE, SDS 0.1% in hybridization temperature. Incubation with conjugated horseradish peroxidase-streptavidin (1 mg/mL; Vector Laboratories, Burlingame, CA, USA) was performed in room temperature. Membranes were washed 2 times with 2 × SSPE, SDS 0.1% for 10 min to eliminate background and minimize misclassification. Positive reactions were detected by enhanced chemiluminescence (ECL Kit, Amersham) through exposure to Kodak X-OMAT film (Kodak, Rochester, NY). If needed to obtain optimal signal intensities, the exposure times were sometimes varied, as judged by evaluation of the hybridization controls present on each membrane.

### Restriction Fragment Length Polymorphism (RFLP)

After *TP53 *exon 4 amplification, 7 μL of the amplified reaction products were digested in a 10 μL volume of 1 × NEB buffer 2 and 5 U of *Bsa*JI or *Bst*UI endonucleases (New England BioLabs, Newton, MA) at 60°C for at least 2 hours. Products digested by *Bsa*JI were run in 12% polyacrylamide gel and products digested by *Bst*UI were run in 8% polyacrylamide gel. In both cases silver staining was used to identify band profile [16].

To ensure quality control of all genotyping methods, the samples were blindly read by two persons independently (TR, LLV). Despite the high percentage of agreement, around 95% for each method, the samples with discordant results were submitted to a new round of amplification and genotyping.

### Statistical Analysis

Cohen's kappa coefficients of agreement were computed to evaluate the inter-methodology variability in genotyping.

## Competing interests

The authors declare that they have no competing interests.

## Authors' contributions

TR performed the analysis, interpreted the data and drafted the manuscript. LLV reviewed the analysis. HT performed the statistical analysis and revised the manuscript. EF and LLV contributed to the design and coordination of the study and revised the manuscript. All authors read and approved the final manuscript.
